# A longitudinal study of physical activity among Malaysian breast cancer survivors

**DOI:** 10.1371/journal.pone.0277982

**Published:** 2022-11-21

**Authors:** Yi Lin Lee, Tania Islam, Mahmoud Danaee, Nur Aishah Taib, on behalf of the MyBCC study group

**Affiliations:** 1 Department of Surgery, Faculty of Medicine, Universiti Malaya, Kuala Lumpur, Malaysia; 2 Centre for Clinical Trial, Institute for Clinical Research, Hospital Ampang, Ampang, Selangor, Malaysia; 3 Universiti Malaya Cancer Research Institute, Kuala Lumpur, Malaysia; 4 Department of Social and Preventive Medicine, Faculty of Medicine, Universiti Malaya, Kuala Lumpur, Malaysia; University of Hertfordshire, UNITED KINGDOM

## Abstract

Regular physical activity (PA) after a breast cancer diagnosis is associated with reduced mortality and better quality of life. In this prospective cohort study, we aimed to explore the trends of PA among breast cancer survivors over three years and identify factors associated with low PA. Interviews on 133 breast cancer patients were conducted at baseline, one and three years after the diagnosis of breast cancer at University Malaya Medical Centre in Kuala Lumpur. Physical activity was measured by using the Global Physical Activity Questionnaire. PA was categorised as *active* (≥ 600 MET-min/week) and *inactive* (<600 MET-min/week). We used the generalised estimating equation method to examine PA levels and factors affecting PA longitudinally. The survivors’ mean age was 56.89 (±10.56) years; half were Chinese (50.4%), and 70.7% were married. At baseline, 48.1% of the patients were active, but the proportion of active patients declined to 39.8% at one year and 35.3% in the third year. The mean total PA decreased significantly from 3503±6838.3 MET-min/week to 1494.0±2679.8 MET-min/week (one year) and 792.5±1364 MET-min/week (three years) (p<0.001). Three years after diagnosis (adjusted odds ratio [AOR]: 1.74, p = 0.021); Malay ethnicity (AOR: 1.86, p = 0.042) and being underweight (AOR: 3.43, p = 0.004) were significantly associated with inactivity. We demonstrated that breast cancer survivors in Malaysia had inadequate PA levels at diagnosis, which decreased over time. Thus, it is vital to communicate about the benefits of PA on cancer outcomes and continue to encourage breast cancer survivors to be physically active throughout the extended survivorship period, especially in the Malay ethnic group and underweight patients.

## Introduction

In Malaysia, breast cancer is the most commonly diagnosed cancer, accounting for 32.7% of newly diagnosed cancer cases among women in 2018 [1]. According to the Malaysia National Cancer Registry Report 2012–2016, the age-standardised rate of breast cancer increased from 31.1 in 2007–2011 to 34.1 in 2012–2016 per 100,000 population [2]. The rise in the incidence and survival rate over the past three decades [3] due to improvement in treatment and early detection could contribute to a growing population of breast cancer survivors. As a result, there has been increased interest in the factors affecting their quality of life (QOL) and survival.

In recent years, physical activity (PA) has consistently been a vital modifying factor in breast cancer. Physical activity improves QOL and reduces mortality in breast cancer survivors by increasing physiological function and modulating metabolic markers [4]. Several studies have found that survivors who have engaged in weight training have a significant increase in upper-lower body strength, grip strength, hip flexibility, and extension [5–7]. Furthermore, aerobic training significantly increased natural killer cells and lymphocytes, which enhanced the immune response [8]. Regular exercise reduces obesity and improves gastrointestinal function, which will positively affect body composition and the overall outlook on life [9]. Given the benefits of PA, the National Cancer Society Malaysia (2019) recommends that 2.5 hours per week of moderate-intensity exercise or 1.25 hours per week of vigorous-intensity aerobic exercise in a combination of 2–3 sessions of resistance training should be the targets for cancer survivors; similar to the recommendations outlined by other international bodies [10–12].

Breast cancer survivors usually have lower PA levels after diagnosis [13] owing to side effects [14] such as pain and upper body weakness [15]. Physical activity levels were also reported to decrease over the follow-up period. In the Health, Eating, Activity, and Lifestyle (HEAL) cohort study, only 21.4% of the subjects met the PA guidelines ten years after enrolment compared to 34% pre-diagnosis [16]. Additionally, two case-control studies conducted on the east coast of Malaysia and Klang Valley found that breast cancer survivors spent lesser time on all activities each year [17] and about half of them had not undertaken any PA throughout their lifetime as compared to the control group(19%) [18]. Another case-control study conducted in the Klang Valley also reported that breast cancer survivors had low-to-moderate levels of PA, while healthy controls participated in moderate-to-vigorous levels of PA [19]. These results suggest that breast cancer patients in Malaysia generally have lower PA levels compared to the healthy population. The effect of a breast cancer diagnosis on long-term PA has not been well researched in Asia. Most prospective studies have been conducted in Western populations except for one cohort study conducted in Hong Kong. Hence, this study aimed to examine longitudinal PA levels and identify associated factors among Malaysian breast cancer patients during the extended survivorship period.

## Method

### Study population

This study is a part of a larger study, the Malaysian Breast Cancer Survivorship Cohort (MyBCC) study [20]. In this study, we examined the physical activity of the patients who had completed three follow-ups at baseline, the 1-year, and 3-years. MyBCC is a hospital-based prospective cohort study to ascertain the relationship between multi-ethnic BC survivors’ socioeconomic level, body composition, lifestyle factors, psychosocial characteristics, return to work, use of complementary and alternative medicine, and overall survival and quality of life [20]. It was conducted at the University of Malaya Medical Centre (UMMC), a tertiary referral centre in the Klang Valley. Subject eligibility included Malaysian women aged 18 years and above with newly diagnosed primary breast cancer in UMMC and recruited within four months of diagnosis. Patients with a prior history of any other cancer, certified as unfit due to other prevailing medical conditions, and bedridden at recruitment were excluded. All study procedures, research purposes, and ethical issues were explained to all participants before obtaining informed consent. Trained interviewers collected the data by administering questionnaires through regular clinic visits in breast cancer outpatient clinics in UMMC and scheduled phone calls. Ethics approval was obtained from the University of Malaya Medical Centre Ethical Committee (MEC number 896.150) for this study. Informed written consents were obtained from the study participants.

### Assessment of physical activity

The Global Physical Activity Questionnaire (GPAQ) (version 2) [21] or its validated Malay version [22] was used to measure the PA levels of the participants. This questionnaire has three domains (work, transportation, and recreational activities) and 16 items, measuring the participant’s amount of time on each activity in a typical week. Examples of activity captured in the work domain include paid or unpaid work, study or training, household chores, harvesting food or crops, fishing or hunting for food and seeking employment. In the transportation domain, PA is defined as activities spent to travel to and from places such as work, shopping, market and place of worship. Recreational activities include sports, fitness and leisure activities [21]. Data cleaning including removing duplicate or irrelevant responses, filtering unwanted outliers and handling missing data were performed according to the WHO GPAQ protocol, version 2 [21].

The amount of time spent on each activity was converted to the metabolic equivalent of the task (METs). One MET is defined as 1 kcal/kg/h, which is equivalent to the energy cost of sitting quietly [21]. Walking and moderate activities were quantified as having 4.0 METS, and vigorous activity was 8.0 METS. The total MET-min/week and intensity of the activities were used for PA level classification. The GPAQ guidelines allowed participants to be grouped into *active* (≥ 600 MET-min/week) or *inactive* (<600 MET-min/week) groups according to the WHO recommendation on physical activity [23]. Active participants were individuals who achieved a minimum of at least 600 MET-min/week. Inactive participants were those who did not fulfil these criteria. Changes in PA levels from baseline were classified as “decreased”, “increased”, or “the same”.

### Sociodemographic factors

Sociodemographic information was collected at baseline using a standardised questionnaire. Weight was measured using a portable body composition analyser (TANITA BC-418 Body Composition Analyser; Tokyo, Japan). Body mass index (BMI) was categorised according to the Clinical Practice Guidelines on the Management of Obesity (Malaysia) [24]. Those with BMI<18.5 kg/m^2^ are considered underweight while BMI>27.4 kg/m^2^ is obese. For marital status, single, widowed, or separated survivors were classified as ‘unmarried’. A patient who has undergone any form of treatment will be coded as yes for the type of treatment they received. Ethnicity was also collected as sociocultural and linguistic norms defer between the multiracial patients in Malaysia, ethnicity was recorded in order to facilitate future targeted interventions.

### Statistical analysis

Descriptive statistics summarised continuous variables, including mean and standard deviation (SD). Frequencies and percentages summarised discrete variables. Tests of differences were performed between the inactive and active groups using the independent t-test for continuous variables and Pearson chi-square or Fisher exact test for categorical variables.

The generalised estimating equation (GEE) method was used to compare the general trend of PA at three-time points. Physical activity levels, which showed significant differences over time, were adjusted for sociodemographic factors. Post-hoc analysis using the sequential Bonferroni method was used to examine the differences between the groups.

Additionally, the factors associated with PA levels in repeated time measurements were analysed using GEE. Physical activity levels were coded as a binary outcome, where the active group was used as the denominator. Independent variables with a p-value cut-off point of <0.25 (based on the Wald test from logistic regression) were first selected using the univariable GEE analyses [25]. The stepwise variable selection method was then used, and models with the lowest corrected quasi-likelihood under the independence model criterion (QICC) were chosen. The model employed an exchangeable correlation structure because it yielded the lowest quasi-likelihood under the independence model criterion (QIC) values. Multicollinearity was not detected in any of the models.

Data cleaning and statistical analysis were performed using R statistical software version 3.6.2. Statistical significance was set at P < 0.05.

The sample size of this study is sufficient to achieve a power of 0.81. The post-hoc power calculation was performed for the association between ethnicity and PA using PASS software version 20.0.6. A two-sided Wald test from a GEE analysis was done to test the proportion difference between Malay and non-Malay inactive survivors at a significance level of 0.05. The proportions of inactive subjects at three years post-diagnosis were 0.79 and 0.58 between Malay and non-Malay respectively. The result showed that a total sample of 133 subjects achieved a power of 0.81.

## Result

### Patient characteristics

One hundred thirty-three women with a mean age of 56.89 (±10.56) years were followed-up at baseline, one year and three years. The details of patient characteristics are shown in Table 1. Approximately half of them were Chinese (50.4%). Most were married (70.7%), had at least a secondary school education (73.7%), and were not working (60.9%). The mean BMI was 27.15 (± 6.31). There were 31.6% overweight and 42.1% obese participants. Most of them (68.4%) were postmenopausal. The clinical characteristics showed that 76.7% of the patients were diagnosed with early-stage disease, 62.4% had undergone a mastectomy, 58.6% had chemotherapy, 62.4% had radiotherapy, and 82.7% had hormonal therapy.

**Table 1:** Background characteristics of the patients at baseline, n = 133.

Characteristics	N (%) or Mean (SD)
Age, years (mean (SD))	56.89 (±10.56)
Age, years	
<50	37 (27.8)
≥50	96 (72.2)
Ethnicity	
Chinese	67 (50.4)
Indian	24 (18.0)
Malay	42 (31.6)
Marital status	
Not married (single/ widowed/ separated)	39 (29.3)
Married	94 (70.7)
Income	
<RM1500	32 (24.1)
RM1500-RM4999	68 (51.1)
≥RM5000	31 (23.3)
Unknown	2 (1.5)
Education	
No formal education/ Primary	32 (24.1)
Secondary	63 (47.4)
University/college	35 (26.3)
Unknown	3 (2.3)
Occupation	
Not working	81 (60.9)
Working	52 (39.1)
Stage	
≤2	102 (76.7)
3&4	31 (23.3)
BMI, kg/m^2^ (mean (SD))	27.15 (6.31)
BMI category, kg/m^2^	
Underweight (<18.5)	7 (5.3)
Normal (18.5–22.9)	28 (21.1)
Overweight (23–27.4)	42 (31.6)
Obese (>27.4)	56 (42.1)
Menopausal status	
Premenopausal women	42 (31.6)
Postmenopausal women	91 (68.4)
Family history of cancer	
No	66 (49.6)
Yes	67 (50.4)
Type of surgery	
Breast-conserving surgery	49 (36.8)
mastectomy	83 (62.4)
no surgery	1 (0.8)
Chemotherapy	
No	55 (41.4)
Yes	78 (58.6)
Radiotherapy	
No	50 (37.6)
Yes	83 (62.4)
Hormonal therapy	
No	23 (17.3)
Yes	110 (82.7)
Recurrence	
No	131 (98.5)
Yes	2 (1.5)

In Table 2, the patients’ background characteristics are compared between the active and inactive groups based on their baseline PA levels. There were no significant differences between the inactive (<600 MET-min/week) and active (≥600 MET-min/week) groups.

**Table 2:** Background characteristics of inactive vs. active patients at baseline, n = 133.

	N (%) or Mean (SD)		
Characteristics	Inactive	Active	p-value^a^
	(<600 MET mins/ week), n = 69	(≥600 MET mins/ week), n = 64	
^b^Age, years (mean (SD))	58.28 (10.71)	55.41 (10.27)	0.117
^**a**^Age, years			0.395
<50	17 (24.6)	20 (31.2)	
50	52 (75.4)	44 (68.8)	
^**a**^Ethnicity			0.539
Chinese	36 (52.2)	31 (48.4)	
Indian	10 (14.5)	14 (21.9)	
Malay	23 (33.3)	19 (29.7)	
^**a**^Marital status			0.291
Not married (single/ widowed/ separated)	23 (33.3)	16 (25.0)	
Married	46 (66.7)	48 (75.0)	
^**a**^Income			0.734
<RM1500	15 (21.7)	17 (26.6)	
RM1500-RM4999	37 (53.6)	31 (48.4)	
≥RM5000	15 (21.7)	16 (25.0)	
Unknown	2 (2.9)	0 (0.0)	
^**a**^Education			0.412
No formal education/ Primary	20 (29.0)	12 (18.8)	
Secondary	31 (44.9)	32 (50.0)	
University/college	17 (24.6)	18 (28.1)	
Unknown	1 (1.4)	2 (3.1)	
^**a**^Occupation			0.077
Not working	47 (68.1)	34 (53.1)	
Working	22 (31.9)	30 (46.9)	
^**a**^Stage			0.973
≤2	53 (76.8)	49 (76.6)	
3&4	16 (23.2)	15 (23.4)	
^b^BMI, kg/m^2^ (mean (SD))	27.53 (7.03)	26.74 (5.45)	0.476
^c^BMI category, kg/m^2^			0.106
Underweight (<18.5)	5 (7.2)	2 (3.1)	
Normal (18.5–22.9)	14 (20.3)	14 (21.9)	
Overweight (23–27.4)	16 (23.2)	26 (40.6)	
Obese (>27.4)	34 (49.3)	22 (34.4)	
^**a**^Menopausal			0.298
Pre	19 (27.5)	23 (35.9)	
Post	50 (72.5)	41 (64.1)	
^**a**^Family history of cancer			0.541
No	36 (52.2)	30 (46.9)	
Yes	33 (47.8)	34 (53.1)	
^c^Type of surgery			0.786
Breast-conserving surgery	24 (34.8)	25 (39.1)	
mastectomy	44 (63.8)	39 (60.9)	
no surgery	1 (1.4)	0 (0.0)	
^**a**^Chemotherapy			0.589
No	27 (39.1)	28 (43.8)	
Yes	42 (60.9)	36 (56.2)	
^**a**^Radiotherapy			0.982
No	26 (37.7)	24 (37.5)	
Yes	43 (62.3)	40 (62.5)	
^**a**^Hormonal therapy			0.975
No	12 (17.4)	11 (17.2)	
Yes	57 (82.6)	53 (82.8)	
^c^Recurrence			1.000
No	68 (98.6)	63 (98.4)	
Yes	1 (1.4)	1 (1.6)	

^a^Chi-square test for independence test was performed

^b^Independent t-test, ^c^Fisher’s exact test, BMI: body mass index, SD: standard deviation

### Physical activity

Physical activity levels reduced over the three years (Table 3). At baseline, 51.9% of patients were inactive, which increased to 60.2% and 64.7% at one and three years, respectively (p = 0.040). The mean total MET-min/week was approximately halved at every time point. The mean total MET-min/week was 3503.0 (±6838.3) at baseline, which decreased to 1494.0 (±2679.8) and 792.5 (±1364.0) (p<0.001). This drastic decrease could be because they reduced work after diagnosis and undergoing treatment, as the work domain dominated at each time point. The mean score in the work domain at baseline was 2379.0 MET-min/week, which was 70% of the PA compared to only 639.2 MET-min/week in the transport domain and 484.8 MET-min/week in the recreation domain. The mean PA in the work domain reduced further to 533.7 MET-min/week at one year and became the most negligible contribution to PA (mean of 139.8 MET-min/week at three years) (p<0.001). The mean PA in the transportation domain also decreased to 518.8 MET-min/week at one year and 268.9 MET-min/week at three years (p = 0.020). For the recreation domain, there was a decline from baseline to three years (mean of 484.8 MET-min/week, 441.1 MET-min/week to 383.8 MET-min/week, respectively), but the differences were not significant (p = 0.488).

**Table 3:** Physical activity levels at baseline, one year and three years.

Physical activity	Baseline, MET-min/ week		One year, MET-min/ week		Three years,MET-min/ week		p-value
	Mean (SD)	Median (IQR)	Mean (SD)	Median (IQR)	Mean (SD)	Median (IQR)	
Total	3503 (6838.3)	960 (2360)	1494.0 (2679.8)	600 (1080)	792.5 (1364.0)	360 (960)	<0.001 ^a^*
Work	2379	0	533.7	0	139.8	0	<0.001 ^b^*
	(6237.2)	(480)	(1601.8)	(40)	(872.5)	(0)	
Transport	639.2	0	518.8	0	268.9	0	0.020 ^c^**
	(1525.5)	(560)	(1744.5)	(360)	(818.8)	(240)	
Recreation	484.8	0	441.1	0	383.8	180	0.488
	(892.3)	(600)	(917.1)	(600)	(573.2)	(560)	
**Level, n (%)**							
Inactive	69 (51.9)		80 (60.2)		86 (64.7)		0.040 ^d^
Active	64 (48.1)		53 (39.8)		47 (35.3)		

GEE with an exchangeable correlation structure was used.

^a^ Adjusted with age, body mass index, occupation, menopausal and radiotherapy

^b^ Adjusted with age, stage, occupation, menopausal and radiotherapy

^c^ Adjusted with age, marital status, income, stage, BMI, menopausal, family history of cancer and type of surgery

^d^ Adjusted with age, body mass index, ethnicity and hormonal therapy

* Sequential Bonferroni post-hoc test showed significance for all groups

** Sequential Bonferroni post-hoc test showed significance for baseline vs three year

Nevertheless, it was observed that 50%-70% of the patients did not have changes in PA levels at any time point (Table 4). The pattern of change was similar for baseline vs three years and one year vs three years. Still, for baseline versus one year, a very low proportion of patients (9.8%) increased their PA levels, and most of them maintained the same level of PA (72.2%).

**Table 4:** Changes of physical activity levels.

Change of Physical Activity	N (%)		
	Decreased	Increased	Same
Baseline vs. one year	24 (18.0)	13 (9.8)	96 (72.2)
One year vs. three years	40 (30.1)	25 (18.8)	68 (51.1)
Baseline vs. three years	36 (27.1)	19 (14.3)	78 (58.6)

### Factors affecting physical activity

The significant predictors for inactivity identified by multivariable regression were; three years after diagnosis (adjusted odds ratio [AOR] 1.74; 95%CI: 1.09, 2.79; p = 0.021), being Malay (AOR 1.86, 95% CI: 1.02, 3.37; p = 0.042) and being underweight (AOR 3.43, 95% CI: 1.49, 7.90; p = 0.004) (Table 5). The GEE model had a QIC of 531.8 and a QICC of 532.3.

**Table 5:** Factors associated with inactivity among breast cancer patients.

	Univariable		Multivariable	
	Odds ratio (95% CI)	p-value	Odds ratio (95% CI)	p-value
(Intercept)			0.69 (0.35, 1.33)	0.271
Age, years				
<50	1.0			
≥50	1.02 (0.58, 1.79)	0.946		
Ethnicity				
Chinese	1.0		1.0	
Indian	1.25 (0.65, 2.41)	0.495	1.15 (0.60, 2.19)	0.676
Malay	2.00 (1.14, 3.49)	0.015	1.86 (1.02, 3.37)	0.042
Time				
Baseline	1.0		1.0	
One year	1.40 (0.98, 2.01)	0.068	1.42 (0.97, 2.08)	0.068
Three years	1.70 (1.08, 2.66)	0.021*	1.74 (1.09, 2.79)	0.021*
Marital status				
Married	1.0			
Not married (single/ widowed/ separated)	1.17 (0.67, 2.05)	0.587		
Education				
No formal education/ Primary	1.0			
Secondary	0.82 (0.44, 1.51)	0.518		
University/college	0.74 (0.37, 1.46)	0.381		
Occupation				
Not working	1.0			
Working	0.88 (0.54, 1.44)	0.618		
Income				
<RM1500	1.0			
RM1500-RM4999	1.20 (0.68, 2.12)	0.521		
≥RM5000	0.87 (0.43, 1.73)	0.686		
Stage				
≤2	1.0			
3&4	1.21 (0.65, 2.26)	0.554		
BMI category, kg/m^2^				
Normal (18.5–22.9)	1.0		1.0	
Underweight (<18.5)	3.20 (1.39, 7.39)	0.006*	3.43 (1.49, 7.90)	0.004*
Overweight (23–27.4)	1.07 (0.53, 2.15)	0.859	0.92 (0.45, 1.88)	0.827
Obese (>27.4)	2.00 (1.01, 3.96)	0.047*	1.57 (0.78, 3.16)	0.210
Menopausal				
Pre	1.0			
Post	1.17 (0.68, 1.99)	0.575		
Family history of cancer				
No	1.0			
Yes	0.91 (0.56, 1.47)	0.690		
Type of surgery				
Breast-conserving surgery	1.0			
Mastectomy	1.15 (0.69, 1.92)	0.595		
Chemotherapy				
No	1.0			
Yes	0.93 (0.57, 1.50)	0.753		
Radiotherapy				
No	1.0			
Yes	0.85 (0.52, 1.39)	0.515		
Hormonal therapy				
No	1.0			
Yes	1.39 (0.78, 2.47)	0.259		

GEE with an exchangeable correlation structure was used. In the multivariable model, no multicollinearity between variables was detected. The model has a QIC of 531.8 and a QICC of 532.3. Significantly different at

*P < .05.

## Discussion

The main findings demonstrated that the breast cancer survivors had low PA levels at all time points. Close to half of the patients (48.1%) were active at baseline. However, PA levels decreased to 39.8% of the patients at one year and 35.3% at three years after diagnosis. Significant declines over the three years were also seen in the total activity, work, and transportation domains. The main reduction was in the work domain where the mean MET-min/week was less than 10% of the baseline. There was no difference in the recreation domain, showing that PA was more related to the work domain and PA related to recreation and exercise was minimal amongst breast cancer survivors in our study. Notably, 70% of the same age bracket general female population in Malaysia are active, this may suggest PA as a risk factor for breast cancer [26, 27]. The predictors of inactivity in our study were duration after diagnosis, Malay ethnicity, and being underweight.

### Physical activity

Previous studies have reported low PA levels in the post-diagnosis period among Caucasian patients (8% to 39.5%) [16, 28, 29] and Asian patients (35% to 39%) [30, 31]. The declining trends in PA are also shown in the cohort studies from Europe and the USA [16, 32–36].

In the immediate period after diagnosis, survivors suffer from treatment-related side effects that negatively impact their PA participation and need to be off work [37, 38]. In our study, the greatest decline in PA was the work domain post-diagnosis, which was consistent with Belgian and American breast cancer survivors [32, 39]. The return-to-work rates were about 43% to 82% in Europe [40–42], 40.6% to 58.9% in Asia [43, 44], and 93% in the USA [45]. To cope with treatment and recovery, our survivors may need to take medical leave, request lighter duties or even opt for early retirement since most of them were reaching the retirement age in Malaysia [44]. The privilege of long medical leave granted to government servant (up to two years) could be another factor [44]. In addition, the diagnosis of BC can cause depression among the survivors which can limit their work productivity including physical tasks [46]. Late stage at presentation is also another barrier of return to work since they would require longer duration of treatment [44]. Concerned friends and family who encourage BC survivors to rest have also caused women to stop doing regular household chores and become less active [13].

Higher rates of return-to-work in other countries have been postulated to be due to employment-related health insurance coverage and marital status [47]. In countries like the USA, the government does not offer free healthcare to the citizens, thus, the survivors will need to continue working to maintain the health insurance coverage provided by the employers [47]. However, in Asia after marriage, women tend to be more committed to their families and most of them are not the principal earners of the family, which may influence them to reduce their focus on work and participate less [47].

Most of the survivors in our study were travelling for work which made up for the PA in the transportation domain. Thus, the reduction in the work domain would have affected the transportation domain. Decreased PA in the transportation domain was also observed in a German study. Post-diagnosis, the breast cancer survivors cycled less for transportation [48]. The decline of social activities post-diagnosis [39, 49] may be another reason for the fall of PA in the transportation domain.

Our finding of non-significance in recreation domains showed that breast cancer patients have low involvement in exercises and sports which is similar to Mason *et al*. They observed a relatively low and constant mean aerobic recreational PA level up to 5 years post-diagnosis [16]. Other cohorts have seen a significant decline in recreational PA compared to baseline [32, 39].

The only Asian cohort study that was conducted in Hong Kong [30], had a significantly higher level of mean post-diagnosis PA (576 MET-mins/week) than the pre-diagnosis level (354 MET-mins/week) [30]. This difference could be explained by the high percentage (48.2%) of Hong Kong patients who improved their PA levels post-diagnosis. In our study, only a tiny proportion of the participants (10%-18%) had increased PA levels after diagnosis. At three years post-diagnosis, the survivors in our study who increased their PA from baseline were primarily Chinese (p = 0.040) and those with a family income of less than RM1500 (~USD 300) (p = 0.010). Survivors with early-stage breast cancer were also more likely to have increased PA at three years than their PA levels at one year. This group of patients may be similar to the Hong Kong study where the dominant ethnicity is Chinese patients with early breast cancer. Those with a lower income would be more likely to continue working after the diagnosis.

### Factors associated with inactivity

A more extended post-diagnosis period (three years) was a significant predictor of inactivity, but this was not observed for the shorter duration at one-year post-diagnosis. Immediately after diagnosis, it was common to see a decline in PA [29, 34, 39, 50] due to the treatment regimen and side effects such as fatigue and mental stress. However, after treatment completion and adaptation to a new lifestyle, the PA level was expected to be improved [16, 29, 34]. The further decrease seen in the third year could be due to dwindling motivation and losing the “teachable moment” effect where after a diagnosis, survivors are likely to have a wake-up call to improve their general health [51]. Furthermore, there are fewer follow-ups and less encouragement from healthcare providers [52]. The mean age of our survivors at diagnosis was 57 years old; three years after the diagnosis, most of them would experience menopause. Menopause was not a predictive factor in our study. Although promotion of PA among menopausal women were more emphasized frequently, post-menopausal women with breast cancer were less likely to be active in Asian setting [31, 48]. In addition to the treatment side effects, hormonal changes during menopause impairs peripheral vascular reserve, causing decline in exercise capability [53]. The Chinese population study reported lower physical activity among post-menopausal women compared to those who are pre-menopausal [54].

The Malay ethnicity was associated with low PA levels in our study. Loh *et al*. (2013) described several factors for the lack of exercise among the Malay ethnic group, including no social companion, body aches, and knowledge barriers [19]. Furthermore, another study reported that elderly Malays were the least active, as they may feel that they are physically fit from the motions of the prayer, which are practised five times a day [55]. Unfortunately, this domain was not in the construct of the questionnaire, as GPAQ has some limitations in capturing the diversity of activities across cultures [56].

Malay women appear to be at an especially high risk of being physically inactive because they observe the teachings of Islam religion and many Muslims believe physical activities are inappropriate for women [57]. They also have a concern about the exposure of *aurat* when exercising in open areas [58]. *Aurat* refers to the observation of both physical and moral decency, going beyond covering oneself in decent clothing [58]. They expressed dislike of joining sports events which involve both genders in open spaces [59].

Our study showed that being underweight was associated with lower PA, although there were only seven underweight patients, and of these, two were active at baseline; and the confidence interval of the odds was also rather wide. Thus, the results should be interpreted cautiously and this association needs to be further explored in a larger cohort. This finding is similar to a population-based study in the USA [60]. Littman *et al*. also observed that women with BMI < 25 kg/m^2^ had more significant reductions in PA from pre-diagnosis to 12 months post-diagnosis than women with BMI ≥ 30 kg/m^2^ [34]. Underweight women probably had a poorer nutritional status, leading to lower muscle mass and less exercise ability. Contrary to our results, more studies have reported that obese women were less likely to be active than normal-weight women [29, 30, 36, 39, 61].

This study had some limitations. Physical activity was measured using the self-reported GPAQ by the participants and thus, may be prone to over-reporting [22, 62, 63]. Nevertheless, the questionnaire was validated and used throughout. Hence, the PA levels measurements at different time points should be consistent. As we derived the findings from an urban single-centre, our result may not perfectly represent the whole population, as the women’s lifestyle in rural areas might be different. Additionally, the instruments used in the main study, MyBCC, (refer to Methods section), were time-consuming and caused participant fatigue and consequently there were subject dropouts. The initial recruitment of the cohort used another questionnaire, thus, excluding quite a number of patients.

Despite these limitations, the current study had several strengths. This prospective cohort study examined the natural progression of PA as with the standard of care of cancer survivorship in Malaysia where no survivorship programmes or intervention that was available. Hence, it allowed for causation analysis, unlike cross-sectional studies. In addition, we used GEE, which can accommodate missing data and control intercorrelations arising from repeated measurements [64].

These findings highlight the need for a survivorship care plan in Malaysia with regards to general health and wellbeing so clinicians and healthcare providers can provide constant encouragement for breast cancer survivors to participate in PA. Personalised, home-based coaching programs can be implemented to motivate the survivors continuously [65]. A simple mobile app for monitoring, goal setting, exercise prescription and positive communication can be explored to retain survivors’ interest and promote adherence [66]. A rehabilitation program for return to work can be introduced to improve PA in the work domain. Furthermore, incidental exercises such as climbing the staircase instead of taking the lift should be taught as it is easier to be incorporated into daily life. Efforts to understand PA barriers and motivations faced by Malay and underweight survivors would be beneficial in promoting PA among them.

## Conclusion

This study provides valuable information on the PA levels of Malaysian breast cancer survivors throughout their survivorship. We found that PA levels declined over time, especially in the work and transportation domains with very little contribution from the recreational domain. Compared to the general population, the proportions of patients meeting the recommended PA levels were low at all time points (35%, 40% and 48%). The duration since diagnosis, Malay ethnicity and being underweight were significantly associated with inactivity. These preliminary findings will provide evidence for targeted interventions in the survivorship period.
